# Simultaneous broadband and high circular dichroism with two-dimensional all-dielectric chiral metasurface

**DOI:** 10.1515/nanoph-2023-0407

**Published:** 2023-10-13

**Authors:** Rui Wang, Chenqian Wang, Ti Sun, Xin Hu, Chinhua Wang

**Affiliations:** School of Optoelectronic Science and Engineering, Soochow University, Suzhou 215006, China; Key Lab of Advanced Optical Manufacturing Technologies of Jiangsu Province & Key Lab of Modern Optical Technologies of Education Ministry of China, Soochow University, Suzhou 215006, China; School of Optoelectronic Science and Engineering & Collaborative Innovation Center of Suzhou Nano Science and Technology, Soochow University, Suzhou 215006, China

**Keywords:** broadband, high circular dichroism, two-dimensional, all-dielectric, chiral metasurface, multipoles

## Abstract

Chiral metasurfaces have great potential in various applications such as polarimetric imaging and biomedical recognition. However, simultaneous broadband and high circular dichroism (CD) with high polarization extinction ratio (PER) remains a challenge. Here, we present a novel approach to realize simultaneous broadband and high CD with high PER in the optical communication band using a two-dimensional all-dielectric chiral metasurface. The structure is formed by a two-level chiral structure of split cross (first-order) and trapezoid-shaped (second-order) of Si nano ribs, respectively, in which constructively coupled first- and second-order of chirality occurs, resulting in the broad chiral response in the far field of multipoles excited by incident light of different chiralities. Theoretical results show that a CD in transmission reaching 0.9 (up to 0.993) and a PER exceeding 20 dB (up to 35 dB) over the entire wavelength range from 1.39 to 1.61 μm can be achieved simultaneously, consistent with the experimental results of CD ∼0.9 and PER of 10 dB (up to 19.7 dB). Our design paves the way for chiral metasurfaces toward practical applications in terms of working bandwidth, high CD and PER as well as integrality of the devices in many fields.

## 1 Introduction

Chiral materials have diverse applications in polarization detection [1], biomedical molecular recognition [2], circular dichroism spectroscopy [3], optical communications [4], and quantum optics [5], owing to their chiral optical response, namely circular dichroism and optical activity. However, the intrinsic small chiro-optical response of natural materials [6], composed of chiral molecules, leads to bulky optical devices with limited applications in ultra-compact devices. To address this issue, chiral metamaterials composed of artificial structures with a size close to or less than one wavelength have been proposed to achieve strong optical chirality, with an improved chiro-optical response of several orders of magnitude compared to natural materials [7]. To date, various chiral structures have been proposed to achieve strong optical chirality, including 3-dimensional (3D), quasi-3D multilayered, and 2-dimensional (2D) structures. In 2009, Gansel et al. demonstrated a broadband circular polarizer comprising 3D gold helix nanowires with a remarkable chiroptical response in the mid-infrared [8]. The average circular dichroism (CD) in the wavelength range of 3.5–7.5 μm was approximately 0.7 with a polarization extinction ratio (PER) of ∼9 dB. To improve the performance (i.e., operation bandwidth, CD, and PER) of chiral metamaterials, a variety of helical nanostructures have been investigated [9–14]. However, the CDs and especially the PERs are compromised over the operating band (usually <0.8 and <10 dB, respectively) due to surface plasmonics intrinsically associated with Ohmic losses in the structures. In addition to helical structures, optical chiral responses have also been observed on a variety of 3D structures, such as slanted split-ring aperture [15], 3D pinwheel [16], chiral stepped nanoaperture [17], and bended split ring resonators [18]. However, these structures have narrow operating bands with a max CD of about 0.6 and a PER of around 8 dB. Quasi-3D multilayered structures, such as dielectric-metal double layers [19], metal-metal double layers [20], dielectric-dielectric double layers [21], and twisted gold nanorods multi-layered [22] were also proposed. The highest measured mean CD in these structures is about 0.7 [19], and PER is ∼22 dB [20] but only at a single wavelength. Furthermore, the complicated layer-by-layer fabrication process and alignment hinder their practical use. Alternatively, 2D chiral metamaterials offer relatively a simple way to realize the chiro-optical response with 2D structures. In 2016, Khanikaev experimentally demonstrated a 2D plasmonic chiral structure, achieving a CD of approximately 0.2 in transmission over a wavelength range from 8.5 to 9.5 μm [23]. The introduction of a dielectric spacer and a reflector can improve the CD of the plasmonic metasurface, in which a maximum CD of 0.7 can be achieved with a Z-shaped silver (Ag) grating metasurface [24] with a methyl methacrylate (PMMA) spacer and Ag backplane or a CD of a ∼0.5 can be obtained over a broad wavelength range of 1.35–1.85 μm [25] by combining different sizes of gold chiral structures, but the PER is low (<7 dB). Recently, 2D plasmonic chiral magnetic mirrors have been proposed [26, 27] as an effective solution to selectively reflect light of one chirality and absorb light of opposite chirality. These chiral mirrors exhibit a CD up to 0.75 with a PER of approximately 10 dB in a narrow spectral range around 1400 nm (with a bandwidth of ∼20 nm) [27]. In addition, metal–insulator–metal-based reflective devices face challenges in terms of integration with other photonic devices and the inherent loss from plasmonic structures. All-dielectric metasurfaces emerge as an effective way to overcome the limitations of plasmonic-related metasurfaces. In 2015, Wu et al. proposed a silicon 2D chiral structure with a CD of approximately 0.7 in a narrow wavelength range of ∼20 nm centered at 4.7 μm [28]. Subsequently, CDs achieved by all-dielectric 2D chiral structures were improved to greater than 0.8 [29] or even 0.9 [30–32], however, it was all limited within a narrow wavelength range due to the inherent limitation of single resonant mode induced from the structures. While broadband CD has also been observed in all-dielectric 2D chiral structures, their performance (CD <0.5, PER <10 dB) [33, 34] over the operation band falls below that of single-wavelength devices. Some devices exhibit a high CD (∼0.7) only at the central wavelength of the operating band, with CD and PER decreasing rapidly as the wavelength deviates from the central wavelength [35–37].

Here, we propose and experimentally demonstrate an efficient all-dielectric 2D chiral metasurface with a simultaneous high CD and PER as well as a broadband wavelength range covering the optical communication band. Specifically, we observe a CD in transmission that surpasses 0.9 (up to 0.993) and a PER that exceeds 20 dB (up to 35 dB) over the entire wavelength range from 1.39 to 1.61 μm. The performance of the proposed 2D structure over the entire broadband is comparable to or exceeds that of a conventional single-wavelength chiral structure, and far outperforms previously reported broadband 2D chiral structures. The strong chiral response of the proposed device originates from the constructive coupling between the first-order and second-order of chiral structures formed by a split cross and trapezoid-shaped Si nano ribs, from which multipoles excited by incident light of different chiralities selectively interfere constructively or destructively. Notably, we also found that for all-dielectric chiral structures based on multipole resonance, there exists a cutoff wavelength for chiral response. Significantly high CD can only be achieved when the wavelength is shorter than the cutoff wavelength to enable the excitation of the magnetic dipole (MD). Our proposed device achieves near-unity CD and high PER over a broad bandwidth, making it suitable for high-efficiency polarimetric imaging, spin encryptors, and chiral sensors. Moreover, the complementary metal-oxide-semiconductor (CMOS) compatible fabrication techniques of the proposed device provide an enormous advantage for industrial manufacturing and on-chip integration toward practical applications.

## 2 Results and discussion

### 2.1 Structure and simulated performance of proposed chiral metasurface

**Figure 1:**
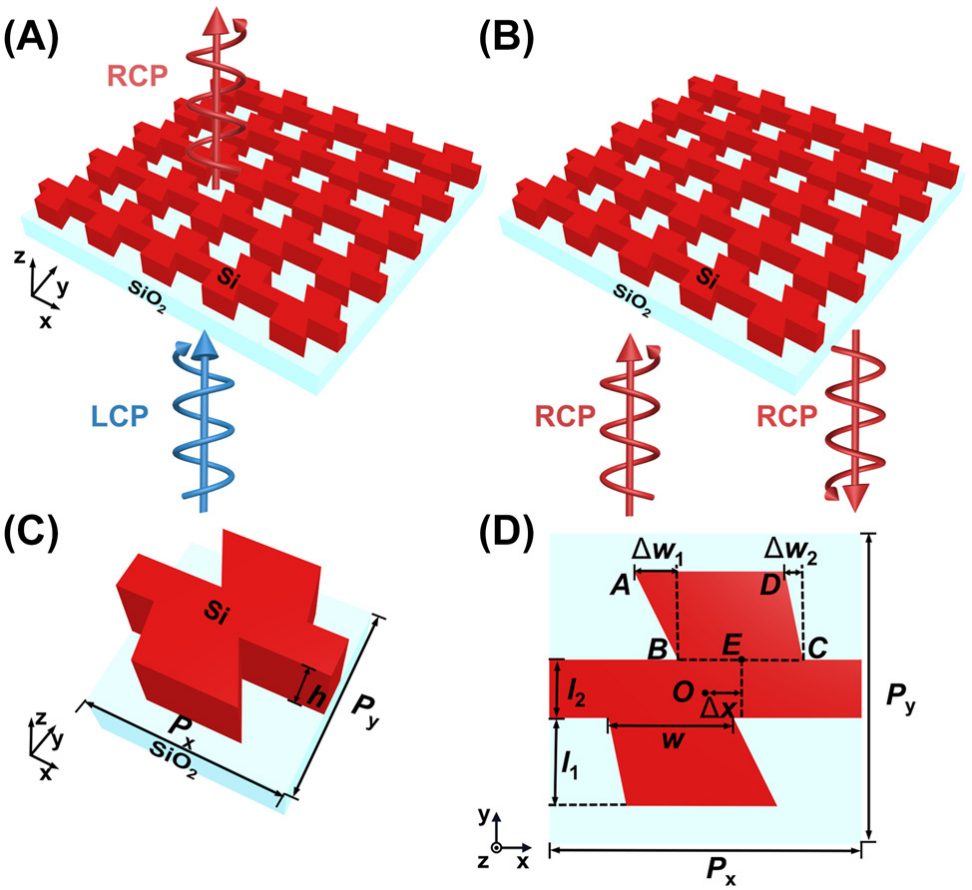
Schematics of a left-handed 2D broadband all-dielectric chiral metasurface. (A, B) Schematics of the chiral responses to LCP and RCP incidences, respectively. (C) Perspective view of a unit cell with chiral a-Si nanopattern on a SiO_2_ substrate. (D) Top view of the unit cell.

Figure 1A and B shows the schematic of the proposed broadband 2D all-dielectric chiral metasurface. The proposed chiral metasurface is formed by an array of the unit cell of split cross amorphous silicon (a-Si) ribs (i.e., first-order chiral structure) with a trapezoid-shaped a-Si wings (i.e., second-order chiral structure or sub-chiral structure) on a SiO_2_ substrate, as depicted in Figure 1C. The period of the unit cell along the *X*- and *Y*-axis is *P*_*x*_, and *P*_*y*_, respectively, and the height of the a-Si ribs is *h*. The first-order chirality of the structure arises from the center (*E*) split of the two trapezoids from the center (*O*) of the unit cell (*O* is the origin of the unit cell) with a distance of Δ*x*, and the second-order chirality (sub-chirality) of the structure is from chiral trapezoids with a width of *l*_1_ in the *y*-direction and a length of *w* connecting to the central bar with a width of *l*_2_, as shown in Figure 1D. The definition of Δ*x* and Δ*w*_1_, Δ*w*_2_ is shown in Figure 1D, where Δ*x* = *x*_*E*_ – *x*_*O*_, Δ*w*_1_ = *x*_*B*_ – *x*_*A*_, Δ*w*_2_ = *x*_*C*_ – *x*_*D*_, where *x*_*E*_, *x*_*O*_, *x*_*A*_, *x*_*B*_, *x*_*C*_, and *x*_*D*_ are the *X*-axis coordinates of points *E*, *O*, *A*, *B*, *C*, and *D*, respectively, and *E* is the midpoint of the lower base of the upper trapezoid *ABCD.* The two trapezoids are assumed to be the same (to simplify the structure) and are symmetric about the origin of the unit cell (*O*), in which a 2-fold rotation (*C*_2_) symmetry of the unit cell (i.e., rotational symmetry about the origin) is formed. It is noted that positive or negative signs of Δ*x* and Δ*w*_1_, Δ*w*_2_ will generate different chiral responses of the structure, in which the sign of Δ*x* will determine the sign of the first-order chirality of the structure while the sign of Δ*w*_1_, Δ*w*_2_ will determine the second-order chirality of the structure. In the following, the structure is assumed to be the first-order “left-handed” or “right-handed” chiral structure when Δ*x* is positive or negative, respectively, and the second-order “left-handed” or “right-handed” chiral structure when Δ*w*_1_ and Δ*w*_2_ are positive or negative, respectively. The selective coupling of the first- and second-order chirality leads to an enhanced CD and PER over a broadband range simultaneously.

Figure 1A and B shows a left-handed circularly polarized (LCP) incidence impinging onto a first-order left-handed chiral metasurface (i.e., Δ*x* is positive), from which a high-efficiency transmission can be obtained with a converted cross circular polarization, i.e., right-handed circularly polarized (RCP). In contrast, when an RCP incidence impinges onto the same metasurface (i.e., the incident circular polarization state is opposed to the helicity of the structure), a high reflection will be obtained with RCP state. It should be noted that the definition of LCP or RCP light is, respectively, anticlockwise or clockwise direction viewed against the direction of light propagation, which is the positive *Z*-axis in transmission or negative *Z*-axis in reflection. The performance of the proposed broadband chiral metasurface was characterized using the finite difference time domain method (FDTD) (Lumerical Finite Difference IDE, Canada). CD and PER in transmission are defined as:

(1) $$\text{CD}=T_{\text{LCP}}-T_{\text{RCP}}$$

(2) $$\text{PER}=10\log\left(T_{\text{LCP}}/T_{\text{RCP}}\right)$$

where *T*_LCP_, *T*_RCP_ represent the transmission of LCP and RCP light. In the calculation, the dielectric properties of a-Si given by Palik [38] are adopted and the refractive index of SiO_2_ is assumed to be 1.44. The two orthogonal circular polarization states can be described by the Jones matrix: ${\vec{\lambda }}_{\text{LCP}}=\frac{1}{\sqrt{2}}\left(1,i\right)$ , ${\vec{\lambda }}_{\text{RCP}}=\frac{1}{\sqrt{2}}\left(1,-i\right)$ .

**Figure 2:**
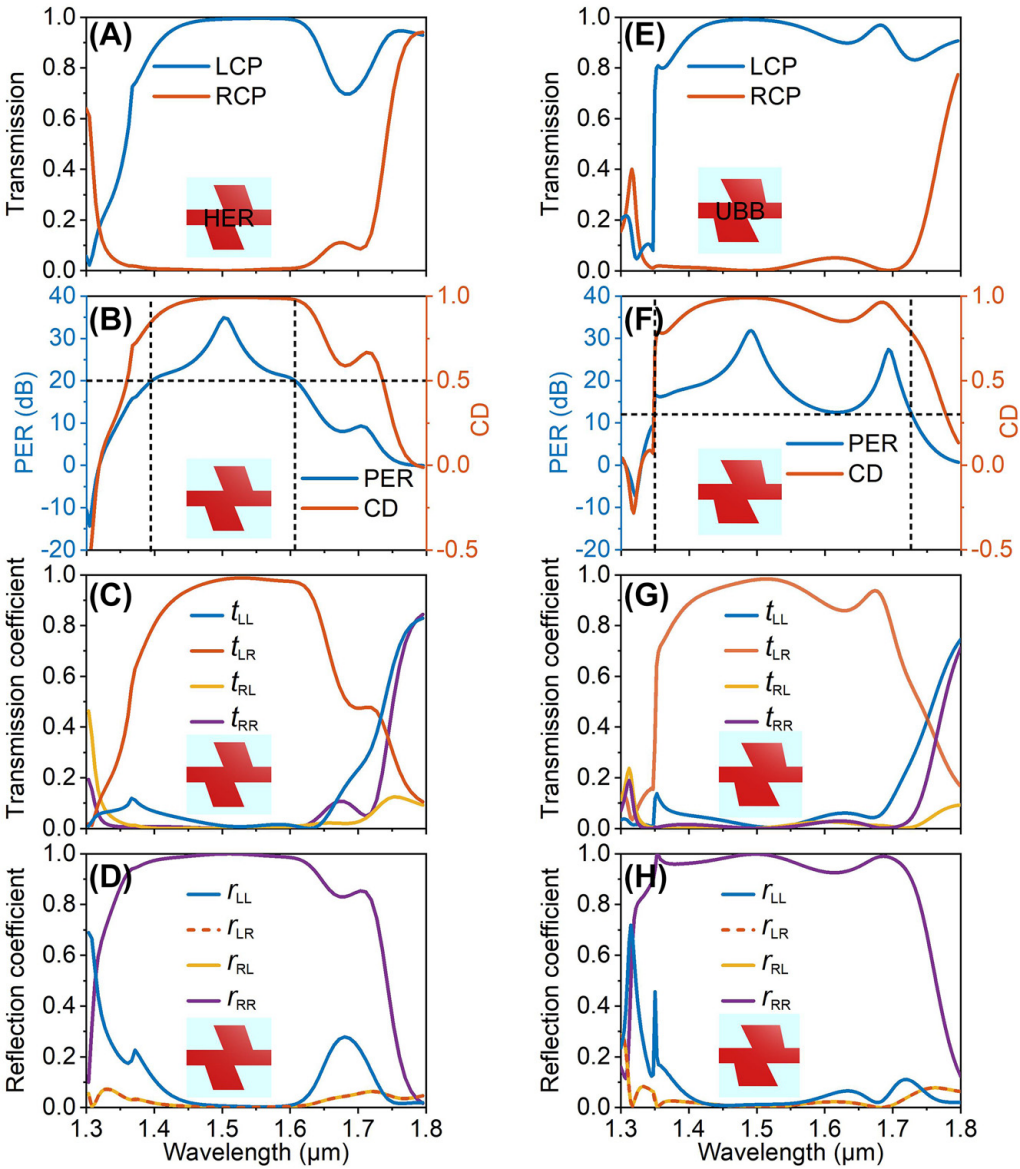
Simulated performance of the proposed 2D chiral nanostructures. (A, E) Transmission spectra of the HER and UBB structure under LCP and RCP illumination. (B, F) The corresponding PER and CD spectra of A, E. (C, G) Transmission coefficient spectra of LCP and RCP components under LCP or RCP incidences for HER and UBB structure. (D, H) Reflection coefficient spectra of LCP and RCP components under LCP or RCP incidences for HER and UBB structure. *t*_*ij*_ denotes the transmittance of “*i*” input and “*j*” output. *r*_*ij*_ denotes the reflectance of “*i*” input and “*j*” output. The subscripts “*L*” and “*R*” represent the LCP and RCP components, respectively.

Figure 2 gives the results of two types of proposed chiral metasurface, in which one structure (labeled as HER, high extinction ratio) shows a very high PER of greater than 20 dB (up to 35 dB) and a high mean CD of greater than 0.9 (up to 0.993) in the wavelength range of 1.39–1.61 μm, as shown in Figure 2A and B, and the other (labeled as UBB, ultrabroadband compared to HER) shows a high extinction ratio of greater than 12 dB (up to 32 dB) and a high mean CD of greater than 0.8 (up to 0.990) in wavelength range of 1.35–1.72 μm, as presented in Figure 2E and F. The corresponding structural parameters for the HER structure are *P*_*x*_ = 947 nm, *P*_*y*_ = 895 nm, *h* = 350 nm, *w =* 330 nm, *l*_1_ = 273 nm, *l*_2_ = 174 nm, Δ*w*_1_ = 111 nm, Δ*w*_2_ = 93 nm, Δ*x* = 105 nm, and *P*_*x*_ = 934 nm, *P*_*y*_ = 900 nm, *h* = 350 nm, *w* = 363 nm, *l*_1_ = 257 nm, *l*_2_ = 165 nm, Δ*w*_1_ = 129 nm, Δ*w*_2_ = 58 nm, Δ*x* = 100 nm for the UBB structure. These optimal parameters are obtained with a particle swarm optimization algorithm in which different target functions are assumed for HER and UBB structures. The details of the optimization and calculation process are given in Figure S1 in Supplementary Material. It is noticed that Δ*x*, Δ*w*_1_, and Δ*w*_2_ are all positive for both HER and UBB structures. Figure 2C and D shows the LCP and RCP components in the transmission and reflection optical fields with both LCP and RCP incidences for the HER structure. It is seen from Figure 2C that the HER structure transmits nearly 100 % of the incident LCP light propagating along +*Z* direction and converts most of the LCP incidence into RCP transmission (*t*_LR_), while highly reflects the RCP incidence light and retains its handedness in the reflection (*r*_RR_), as shown in Figure 2D. Similar to HER, structure UBB highly transmits the incident LCP light propagating along +*Z* direction and converts most of the LCP incidence into RCP transmission (*t*_LR_), while highly reflects the RCP incidence light and retains its handedness in the reflection (*r*_RR_) over a broader band than that of HER as shown in Figure 2G and H. This giant optical chiral response is distinct from that of 3D chiral structures [8, 9, 12] in terms of conversion efficiency of the cross polarization, which is consistent with that observed in *C*_2_ rotation symmetric structures [32, 39] in contrast to structures with 4-fold (*C*_4_) [31] or 3-fold (*C*_3_) rotation symmetry. These results exhibit the possibility of simultaneous broadband, high CD, and high PER with a relatively simple 2D all-dielectric structure, which is comparable to those working at narrow band [30, 32, 39, 40] or single wavelength [41, 42], and superior to all those working in the broadband, to the best of our knowledge.

### 2.2 Interaction of the first- and second-order chirality

**Figure 3:**
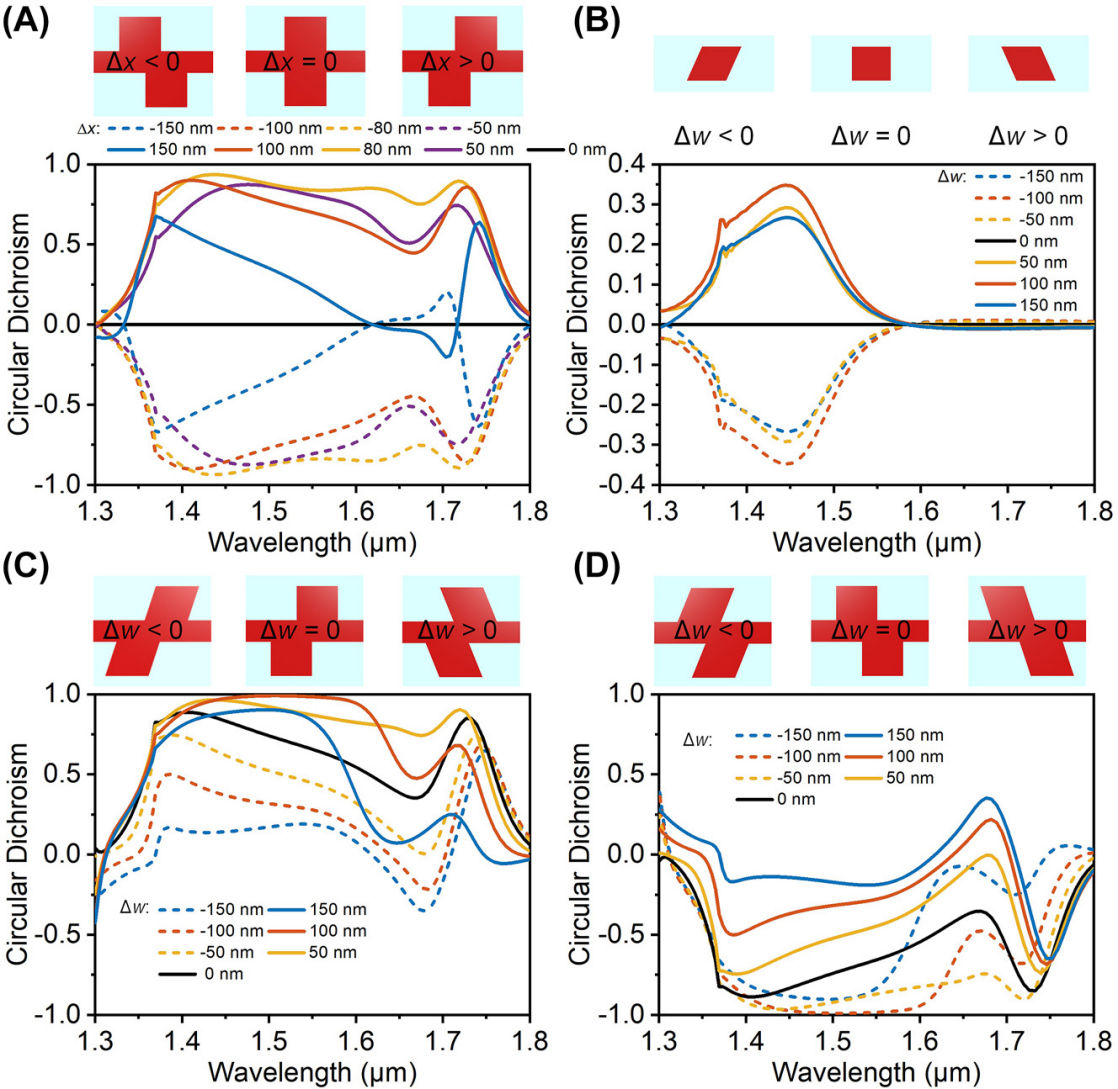
Interaction between the first-order and second-order of chiral structures. (A) CD spectra of the first-order chiral structure as a function of Δ*x*. (B) CD spectra of the second-order chiral structure (parallelogram) as a function of Δ*w*. (C) Overall CD spectra when a first-order left-handed chiral structure with Δ*x* = 105 nm interacts with a second-order chiral structure with different Δ*w*. (D) Overall CD spectra when a first-order right-handed chiral structure with Δ*x* = −105 nm interacts with a second-order chiral structure with different Δ*w*. Other structural parameters are the same as those in the left-handed HER structure in Figure 2A.

The simultaneous broadband, high CD, and high PER with a simple 2D all-dielectric structure can be understood by the constructive coupling between the first- and second-order of chiral structures. Figure 3A shows the CD resulting from the first-order chiral structure by replacing the trapezoid in the HER structure with a rectangle (i.e., Δ*w*_1_ = Δ*w*_2_ = Δ*w* = 0 nm so that second-order chiral structure disappears) while keeping other structural parameters the same as those in Figure 2A. Strong optical chiral responses over a broad wavelength range (1.35–1.75 μm) can be observed when the relative position of the upper and lower rectangles, Δ*x*, is tuned, in which opposite CD responses that correspond to the left-handed and right-handed structures are observed for positive and negative Δ*x*, respectively. The effect of the second-order chiral structure, i.e., the chiral trapezoid wing is also investigated by assuming Δ*w*_1_ = Δ*w*_2_ = Δ*w* and keeping *w* and *l*_1_ the same as those in the HER structure, Figure 3B. The period in the *Y*-axis direction becomes half of that of the HER structure considering the fact that only one trapezoid is used while the period in the *X*-axis direction remains unchanged. It is seen that the nano parallelogram also exhibits a chiral response in 1.35–1.55 μm wavelength range, as shown in Figure 3B, although it is a bit smaller than that of the first-order chiral structure, Figure 3A. In Figure 3B, it is not surprising that no CD can be observed when Δ*w* is zero because of the disappearance of the chirality of the rectangular structure. A positive CD is obtained when Δ*w* is positive, i.e., a left-handed second-order chiral structure as defined above while a negative CD is obtained when Δ*w* is negative, i.e., a right-handed second-order chiral structure.

Figure 3C and D demonstrates the interaction of the first- and second-order chiral structures with different chirality, in which Figure 3C is of left-handed first-order chiral structure, i.e., Δ*x >* 0 (Δ*x* = 105 nm, the same as that in the HER structure in Figure 2A) while the second-order chirality changes with different signs of Δ*w* of the chiral parallelogram, and Figure 3D is of right-handed first-order chiral structure, i.e., Δ*x <* 0 (Δ*x* = −105 nm is assumed) while the second-order chirality changes with different signs of Δ*w* of the chiral parallelogram. It is seen from both Figure 3C and D that the same chirality of a first-order chiral structure and a second-order chiral structure, i.e., Δ*x* and Δ*w* have the same signs (either positive or negative), will significantly enhance the CD on the working band, and opposite chirality between a first-order chiral structure and a second-order chiral structure, i.e., Δ*x* and Δ*w* have opposite signs, will diminish significantly the CD on the band. The optimal coupling is achieved when Δ*w* = 100 nm and Δ*x =* 105 nm in Figure 3C with a maximum near-unity positive CD (solid red line) and Δ*w* = −100 nm and Δ*x =* −105 nm in Figure 3D with a maximum negative CD (dashed red line). Therefore, the constructive coupling between the first- and second-order chirality of the structure renders the solid physics basis for the superior CD performance of the proposed 2D chiral structure.

### 2.3 Multipole decomposition of the chiral optical fields

The underlying physical mechanism for the simultaneous broadband and high CD can also be understood semi-quantitatively with multipole decomposition analysis of the electric field generated from the chiral structure under both LCP or RCP illuminations. The near electric field generated by the chiral structure can be decomposed of multipole moment, in which the first four multipoles are electric dipole moment (ED, labeled with **p**), magnetic dipole moment (MD, labeled with **m**), electric quadrupole moment (EQ, labeled with ${\hat{Q}}^{e}$ , which is a tensor) and magnetic quadrupole moment (MQ, labeled with and ${\hat{Q}}^{m}$ , a tensor), respectively. The far-field transmission or reflection can then be calculated by the contribution from the scattering fields of these multiple components. Detailed information regarding the multipole calculation is given in Equations S2–S5 in Supplementary Material. It is noted that higher order multipoles are not considered because of the negligible contributions (about two orders of magnitude lower than the first four multipoles), as can be seen in Figure S2 in Supplementary Material. To show how these scattered fields of the multipoles contribute to the broadband CD exhibited by the HER metasurface, the backward scattering field, i.e., the reflection electric field, from the first four multipole components is examined [43]:

(3) $$\begin{array}{ll} E_{x}^{\mathrm{sc}} & ∼\left(p_{x}-\frac{1}{v_{d}}m_{y}+\frac{ik_{d}}{6}Q_{xz}^{e}-\frac{ik_{d}}{2v_{d}}Q_{yz}^{m}\right)\\ & =F_{1}\left(p_{x}\right)+{\text{F}}_{2}\left(m_{y}\right)+F_{3}\left(Q_{xz}^{e}\right)+F_{4}\left(Q_{yz}^{m}\right) \end{array}$$

(4) $$\begin{array}{ll} E_{y}^{\text{sc}} & ∼\left(p_{y}+\frac{1}{v_{d}}m_{x}+\frac{ik_{d}}{6}Q_{yz}^{e}+\frac{ik_{d}}{2v_{d}}Q_{xz}^{m}\right)\\ & =F_{5}\left(p_{y}\right)+F_{6}\left(m_{x}\right)+F_{7}\left(Q_{yz}^{e}\right)+F_{8}\left(Q_{xz}^{m}\right) \end{array}$$

where *v*_*d*_, *k*_*d*_ are the speed and wave number of light propagation in the surroundings (air), respectively, and *F*_*i*_ (⋅) (*i* = 1, … 8) represents the scattered vectorial optical fields from the multipole *i*, with phase *φ*_*i*_ = arg(*F*_*i*_ (⋅)). The intensity of the far field of reflection can thus be calculated by ( $|E_{x}^{sc}{|}^{2}+|E_{y}^{sc}{|}^{2}$ ), in which vectorial superposition of the components (*F*_*i*_ (⋅)) from the multipoles is employed.

**Figure 4:**
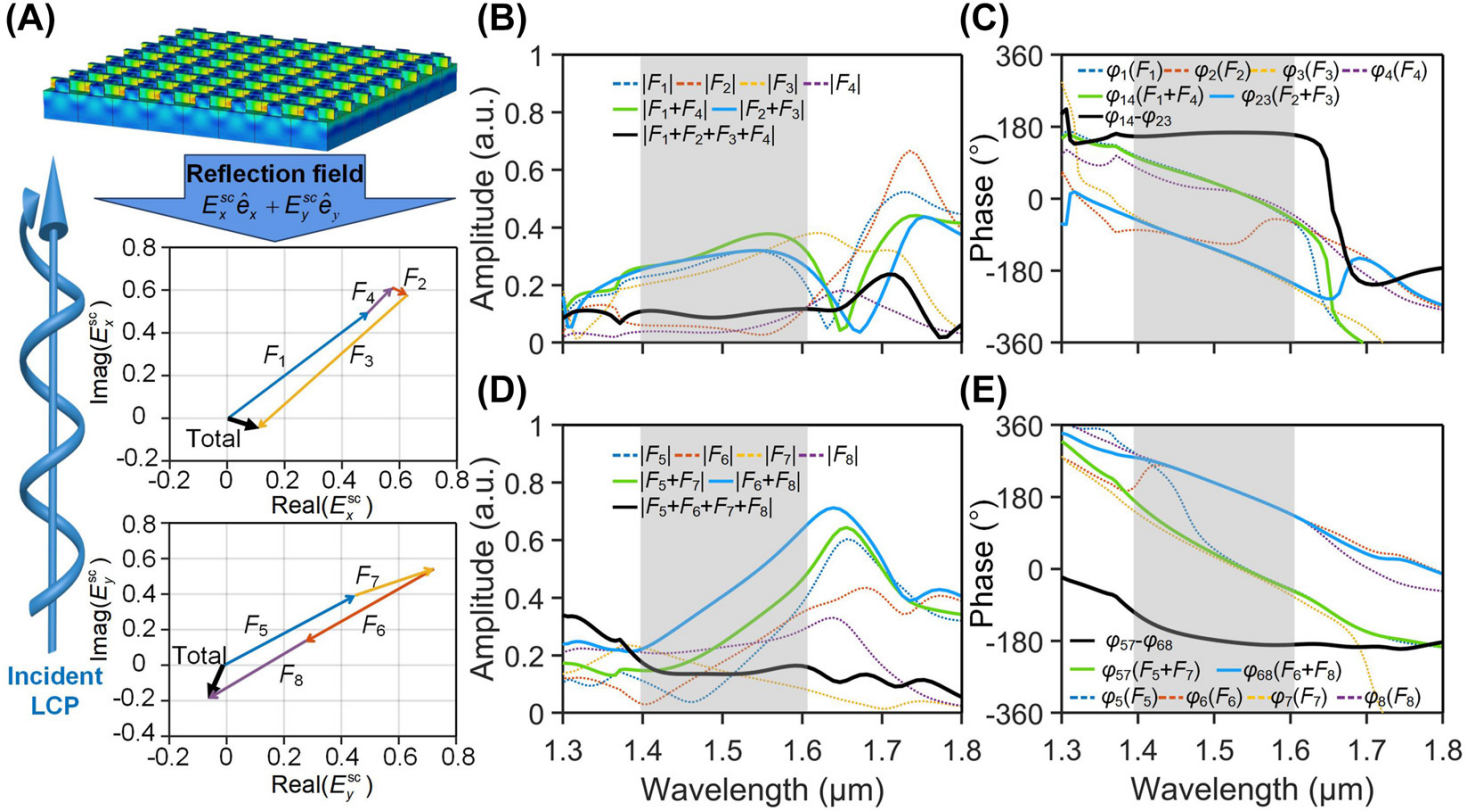
Vectorial field interference of multipoles in the reflection of HER structure under LCP incidence. (A) Illustration of the incident and reflection with HER metasurface and the multipolar interference processes in reflection field under LCP incidence. The vectorial superposition process of the *x*-component ( $E_{x}^{sc}$ ) and *y*-component ( $E_{y}^{sc}$ ) of the four multipoles (*F*_1_–*F*_4_ and *F*_5_–*F*_8_) are given in the complex plane, respectively. (B, D) Amplitudes of the four components of $E_{x}^{sc}$ and $E_{y}^{sc}$ , respectively, as well as the amplitudes formed by the first-stage and second-stage interference. (C, E) The corresponding phases of different components of $E_{x}^{sc}$ and $E_{y}^{sc}$ , and those after the first-stage and second-stage interference.

Figure 4A–E gives the plots of amplitude and phase of these individual components, *F*_*i*_ (⋅) and their interaction process under LCP incidence. The detailed calculation formulas are given in Equations S2–S5 in Supplementary Material. The interaction among the components *F*_*i*_ (⋅) (*i* = 1–4) in $E_{x}^{sc}$ or *F*_*i*_ (⋅) (*i* = 5–8) in $E_{y}^{sc}$ can be understood by interference of these components, as illustrated in Figure 4A. In $E_{x}^{sc}$ (or $E_{y}^{sc}$ ) (Figure 4A), the interferometric field of *F*_1_ + *F*_4_ (or *F*_5_ + *F*_7_) and *F*_2_ + *F*_3_ (or *F*_6_ + *F*_8_) are essentially out of phase, i.e., the phase difference between *F*_1_ + *F*_4_ (or *F*_5_ + *F*_7_) and *F*_2_ + *F*_3_ (or *F*_6_ + *F*_8_) is nearly 180°, which results in destructive interference, and the total interferometric field of the four multipoles is supressed (as indicated with black arrow line in Figure 4A). The detailed amplitudes and phases of the interference process are given in Figure 4B and C, in which the amplitude and phase of the first-stage interference, *F*_1_ + *F*_4_ and *F*_2_ + *F*_3_, are plotted with a solid green and a blue line, respectively, in Figure 4B and C. The amplitude after the second-stage interference between the fields *F*_1_ + *F*_4_ and *F*_2_ + *F*_3_ is plotted with a solid black line in Figure 4B. It is seen that the overall amplitude of $E_{x}^{sc}$ (solid black line) after the 2-stage interference becomes low and flat within the whole wavelength band of interest (grey shaded area) although the amplitudes of *F*_1_ + *F*_4_ and *F*_2_ + *F*_3_ before the 2nd-stage interference are relatively high. The destructive interference that occurred in the 2nd-stage interference is clearly supported by the broad and flat phase difference of 180° between the fields of *F*_1_ + *F*_4_ and *F*_2_ + *F*_3_ in Figure 4C, solid black line. A similar two-stage interference process can also be observed among the four components, *F*_*i*_ (⋅) (*i* = 5–8) in $E_{y}^{sc}$ , as depicted in Figure 4D and E, in which a low and flat amplitude within the interested wavelength band can also be obtained (solid black line in Figure 4D) due to the destructive interference originating from −180° phase difference between *F*_5_ + *F*_7_ and *F*_6_ + *F*_8_ (solid black line in Figure 4E). The intensity of the far field of reflection can thus be calculated by ( $|E_{x}^{sc}{|}^{2}+|E_{y}^{sc}{|}^{2}$ ), which will result in low reflection in the case of LCP incidence. This is well consistent with that observed in Figure 2D in which reflection coefficients of both components (*r*_LL_ and *r*_LR_) are low in the case of LCP incidence. Similarly, the interference process among the multipoles of the HER structure under RCP incidence can be also implemented. The detailed results and figures are given in Figure S3 in Supplementary Material. In contrast to the LCP incidence, a strong reflective field can be obtained for RCP incidence due to the constructive interference, as detailed in Figure S3. The different reflection behaviors between LCP and RCP incidences lead to a substantial difference in the reflectivity of the HER device, thereby giving rise to a large and broadband CD. For the UBB structure, the same interference process can be observed at a wider bandwidth as shown in Figures S5 and S6. The results of scattering CD spectrum based on the above semi-quantitative multipole model for HER and UBB are given in Figures S4 and S7 (Supplementary Material), respectively, which is in agreement with that demonstrated in Figure 2D and H.

**Figure 5:**
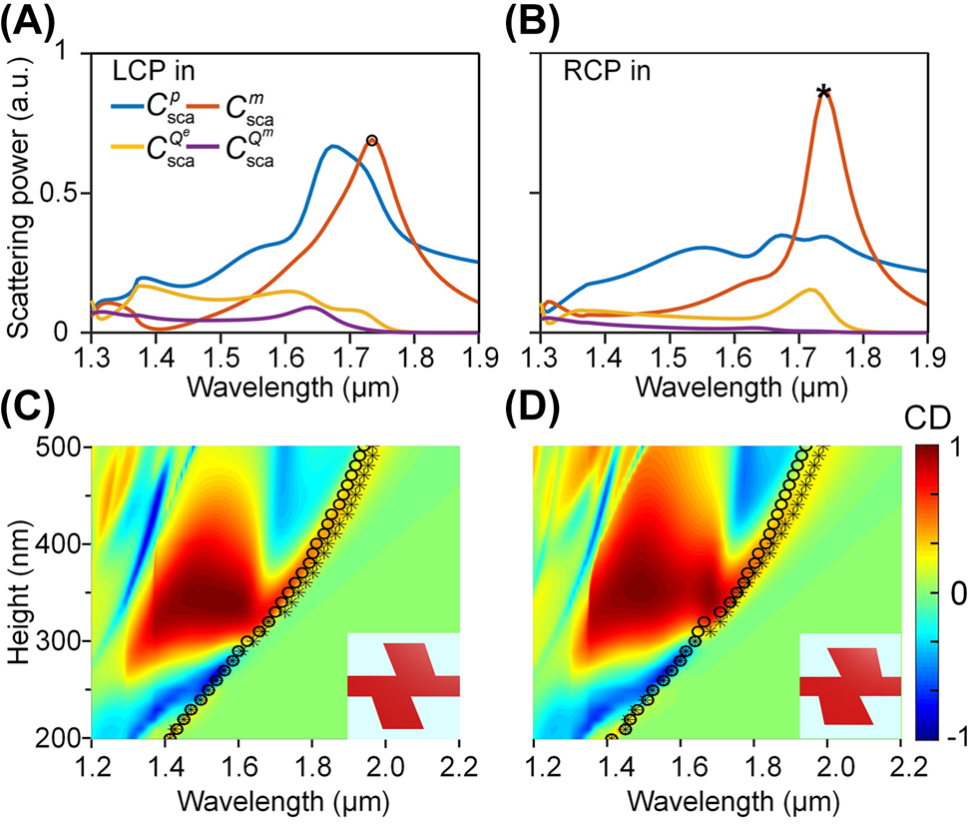
The cutoff behavior of chiral response of the proposed 2D all-dielectric chiral metasurface. Scattering power of individual multipole components in HER structure excited by (A) LCP and (B) RCP incidence. CD maps versus incident wavelength and height of structure of (C) HER structure and (D) UBB structure. Symbols “o” and “*” on the map denote the resonant wavelengths of the MD at the corresponding structure height for LCP and RCP incidence, respectively.

The cutoff behavior of the chiral response in the proposed lossless all-dielectric 2D chiral structure can also be well predicted with the multipoles. Figure 5A and B shows the scattered power ( $C_{\text{sca}}^{i}$ , *i* = **p**, **m**, ${\hat{Q}}^{e}$ , and ${\hat{Q}}^{m}$ , which are given in Equations S7–S10 in Supplementary Material) of ED, MD, EQ, and MQ under LCP and RCP incidences, respectively. The parameters of the HER structure are used in which the height of the structure is *h* = 350 nm. It is seen that MD resonances are excited by both LCP and RCP incidences on the HER structure at wavelengths of 1.73 μm and 1.74 μm, respectively, as indicated by ‘o’ and ‘*’ in Figure 5A and B. Further examination of the MD resonances at different heights (*h*) of the proposed structure shows that the MD resonant wavelength at different heights of the structure forms the cutoff boundary of the chiral response of the proposed 2D chiral structure. Figure 5C and D gives the CD maps versus incident wavelength and the height of the HER and UBB structures (calculated directly with FDTD), respectively, in which symbols ‘o’ and ‘*’ denote the resonance wavelengths of the MD at the corresponding height (calculated with the multipole model as that in Figure 5A and B) under LCP and RCP incidences, respectively. A cutoff boundary of the chiral response in both the CD maps of HER and UBB is observed. Beyond the boundary (i.e., in the region of wavelength longer than the cutoff wavelength), the metasurface exhibits essentially no CD response (<0.1), as shown in the green area on the right side of Figure 5C and D. Remarkably, this boundary aligns excellently with the height-dependent trajectory of the wavelength of the MD resonance excited under both LCP and RCP incidence. Therefore, it is inferred that a cutoff wavelength of the chiral response exists in this type of 2D chiral structure and the cutoff wavelength can be predicted by the MD resonances. This is important in the designing of 2D chiral metasurfaces in which the cutoff wavelength and the corresponding structural dimensions can be pre-identified. The physical mechanism under the observation can be explained with Rosenfeld criterion [31]. For metasurfaces to achieve an optical chiral response, magnetic and electric dipole moments (**p** or **m**) must couple each other, following the Rosenfeld criterion **p**⋅**m** ≠ 0. When the wavelength of incident light is longer than the MD resonance wavelength, no in-plane magnetic dipole forms and therefore, no chiral response occurs. In contrast, when the incident wavelength is shorter than MD resonance wavelength, both the in-plane magnetic dipole and higher-order multipoles are excited, which allows the Rosenfeld criterion satisfied and subsequently the circular dichroism generated. Hence, the MD resonance directly determines the long-wavelength band edge of the CD spectrum.

### 2.4 Measured performance of the fabricated chiral metasurfaces

**Figure 6:**
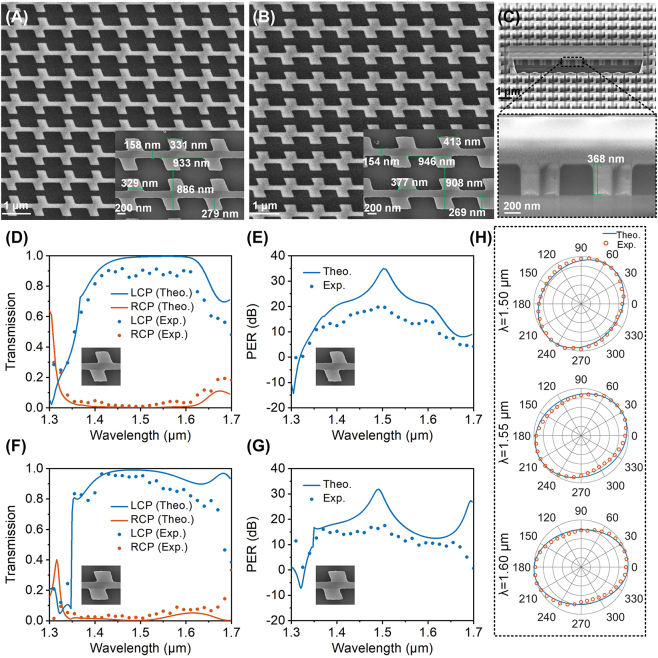
Experimental measurement results. (A) SEM images of the HER. (B) SEM images of the UBB. (C) Typical SEM image of the cross section of the HER. (D) Experimental transmission spectra of HER structure under LCP and RCP illumination. (E) Corresponding PER spectra of HER. (F) Experimental transmission spectra of UBB structure under LCP and RCP illumination. (G) Corresponding PER spectra of UBB. (H) Polarization state of transmitted light after a rotated linear polarizer under LCP incidence onto HER structure at different wavelengths. The distance from the point in the polar coordinate to the origin represents the normalized amplitude.

The scanning electron micrographs (SEM) of the fabricated HER and UBB chiral structure arrays are shown in Figure 6A–C, in which Figure 6A and B is the top views of the HER and UBB structure, respectively, and Figure 6C is a typical cross-section of HER structure. It is seen that the dimensions of the fabricated structures are all in excellent agreement with the designed ones (Figure 2) with a deviation of ±20 nm. Figure 6D–G gives the measured transmittance and PER and their comparison with the theoretical results (solid lines) for the HER and UBB chiral structures, respectively. The measurement setup is given in Figure S8 in Supplementary Material. It is seen that the experimental results are all in good agreement with the simulated results. For the HER structure, Figure 6D and E, the measured average CD in 1.39–1.61 μm is about 0.9, and the average PER in this band is greater than 15 dB, up to 19.7 dB (about 93). For the UBB structure, Figure 6F and G, the measured average CD in 1.35–1.65 μm band is about 0.8, and the measured average PER in this band is greater than 13 dB, up to 17.5 dB (about 56). In order to further verify the polarization characteristics of the transmitted field, a linear polarizer between the CCD detector and the objective lens (O) is added to the measurement setup in Figure S8 (Supplementary Material), and the transmission is measured when rotating the added linear polarizer for HER structure. The measured transmissions and their comparison with the theoretical results at three different wavelengths (1.50 μm, 1.55 μm, and 1.60 μm) are given in a polar coordinate in Figure 6H. It is seen that the experimental and simulation results are all in good agreement at different wavelengths, in which a high ellipticity in the transmission in the wavelength range of 1.50–1.60 μm can be observed due to the high cross-polarization conversion rate of the device, as witnessed in Figure 2C. Compared with recent advances (detailed comparison is given in Table S1 in the Supplementary Material), our proposed structure can simultaneously have a high CD and PER over a broadband wavelength range, which has great potential applications in various polarimetric imaging and detections.

**Figure 7:**
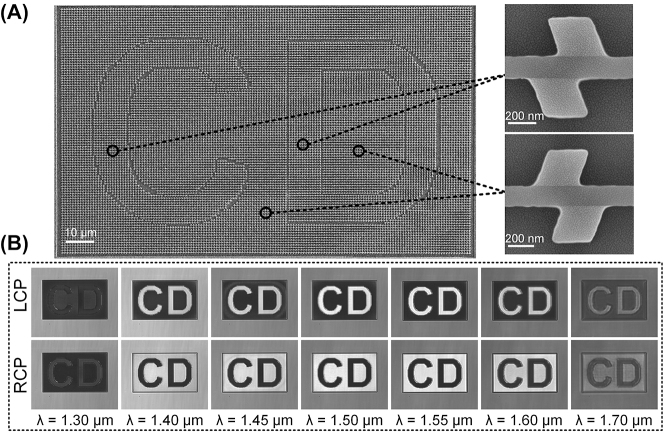
Experimental transmission of the fabricated pattern composed of opposite enantiomers upon circularly polarized illumination. (A) SEM images of the fabricated pattern composed of two opposite chiral enantiomers. The interior and exterior of the letters “CD” are composed of enantiomers of HER, respectively. (B) Experimental images of the transmission of the pattern under the illumination of LCP and RCP at different wavelengths.

To showcase the exceptional performance of proposed chiral structures using a limited number of meta-atoms, we further fabricated a chiral pattern as depicted in Figure 7A, in which the interior and exterior of the letters “CD” comprise enantiomeric counterparts (i.e., opposite chirality) of HER structure, respectively. Figure 7A shows the SEM image of the fabricated “CD” letters composed of two opposite chiral enantiomers inside and outside of the letters, and the corresponding chiral enantiomers are shown on the right side of Figure 7A. The transmissions of the fabricated “CD” pattern under LCP and RCP incidences are shown in Figure 7B in the wavelength range from 1.30 μm to 1.70 μm. In the experiment, to suppress the spatial coherence of the incident beam, a rotating glass diffuser was inserted between the linear polarizer and the attenuator in Figure S8 (Supplementary Material). Remarkably, under LCP illumination within the wide wavelength range of 1.40–1.60 μm, bright letters appear vividly on the dark background continuously, while the letters appear dark on the bright background under RCP illumination. When the incident wavelength deviates from the designed band, the contrast between the background and letters diminishes, as shown in Figure 7B. Especially, contrast reverse phenomenon of the “CD” pattern can be observed at the wavelength of 1.30 μm, which is well consistent with the theoretical prediction shown in Figure 2A as well as the experimental results in Figure 6D. It is noted that the letters’ line width measures around 10 μm, which holds great potential for spin-encoded imaging applications.

## 3 Conclusions

We present a novel all-dielectric 2D chiral structure with the ability to produce broadband chiral response in near infrared wavelength range. The proposed structure is formed by a coupled two-level chiral structure, i.e., a first-order split cross chiral structure and a second-order trapezoid-shaped Si nano ribs, respectively, in which constructive coupling of the first- and second-order of chirality occurs, leading to high levels of CD and PER across a broad spectral range. The observed superior behaviors with different incident chiralities can be well explained with the field interference of multipoles, and the multipole resonance spectrum and CD spectrum at varying heights of the meta-atom reveal the existence of a cutoff wavelength for the optical chiral response of the proposed structure. Beyond this cutoff wavelength, the chiral response is negligible (CD < 0.1), and the cutoff wavelength coincides with the resonance wavelength of the magnetic dipole. The proposed structure and its superior performance has a significant impact on a wide range of applications, including polarimetric imaging, compact polarization detection, secure optical communication systems, circular dichroism spectroscopy and others.

## 4 Methods

The proposed chiral metasurface is fabricated by a multistep lithography process including deposition, patterning, and etching. First, a 1 mm thick, 1.5 × 1.5 cm^2^ piece of optical glass was cleaned as a substrate. A a-Si film with a thickness of 350 nm was deposited onto the substrate by plasma-enhanced chemical vapor deposition (PECVD) at a temperature of 350 °C and a pressure of 1800 mTorr. Then a 360 nm-thick ZEP 520A film was spinning coated as an electron-beam photoresist. An AR-PC 5090 film was spinning coated subsequently as a conductive layer to avoid charge accumulation effect caused by insulating substrates. Electron beam lithography with a current of 2 nA was carried out for the desired pattern exposure. After exposure, the conductive layer was washed away, and then the sample was developed in ZED-N50 for 90 s. The etching process was carried out using inductively couple plasma (ICP) at room temperature for 32 s with a source power of 1200 W, a pressure of 75 mTorr, and gas flow rates of 10 and 30 sccm for sulfur hexafluoride (SF_6_) and trifluoromethane (CHF_3_) respectively. After etching, the residual photoresist was removed using butanone and anhydrous ethanol to complete the metasurface. Using the conventional silicon-based photonic device fabrication process described above, arrays with an area of about 150 μm × 150 μm HER structure and UBB structure were obtained.

## Supplementary Material

Supplementary Material Details
