# Supplementary material for: Simultaneous broadband and high circular dichroism with two-dimensional all-dielectric chiral metasurface
Source: Nanophotonics. 2023 Oct 13;12(21):4043–53. doi: 10.1515/nanoph-2023-0407 (PMC11501280; doi:10.1515/nanoph-2023-0407)
Supplement: Supplementary file 1 — Supplementary Material Details [file j_nanoph-2023-0407_suppl_001.docx]

Supplementary Material

**Simultaneous Broadband and High Circular Dichroism with Two-Dimensional All-Dielectric Chiral Metasurface**

Rui Wang, Chenqian Wang, Ti Sun, Xin Hu*, and Chinhua Wang*

**Corresponding author:** Hu Xin, and Chinhua Wang, School of Optoelectronic Science and Engineering & Collaborative Innovation Center of Suzhou Nano Science and Technology Soochow University, Suzhou 215006, China; and Key Lab of Advanced Optical Manufacturing Technologies of Jiangsu Province & Key Lab of Modern Optical Technologies of Education Ministry of China, Soochow University, Suzhou 215006, China; E-mail: huxin@suda.edu.cn, and chinhua.wang@suda.edu.cn

**Rui Wang, Chenqian Wang, and Ti Sun:** School of Optoelectronic Science and Engineering Soochow University, Suzhou 215006, China; and Key Lab of Advanced Optical Manufacturing Technologies of Jiangsu Province & Key Lab of Modern Optical Technologies of Education Ministry of China, Soochow University, Suzhou 215006, China; E-mail: 20224039003@stu.suda.edu.cn

**1. Details of the optimization and calculation process of the HER and UBB structures**

The structural parameters of the proposed two-dimensional all-dielectric chiral metasurfaces are optimized by the particle swarm optimization (PSO) algorithm, and the process is shown in Figure S1. In order to obtain high circular dichroism (CD) and polarization extinction ratio (PER) simultaneously over the target bands, the figure of merit (*FOM*) is defined as:

$FOM\text{=}A\sum\left(T_{\text{LCP}}-T_{\text{RCP}}\right)+B\sum\left(T_{\text{LCP}}-T_{\text{RCP}}\right)\text{/}\left(T_{\text{LCP}}\text{+}T_{\text{RCP}}\right)$ (S1)

where *T*_LCP_ and *T*_RCP_ are the transmittance matrices of LCP and RCP incident light in the targeted wavelength range, respectively. *A* and *B* are the weighing factors. For the HER structure and UBB structure, the target wavelength range and the weighting factors are set as 1.40–1.60 μm, *A* = 1, *B* = 2 and 1.35–1.75 μm, *A* = 1, and *B* = 1, respectively.


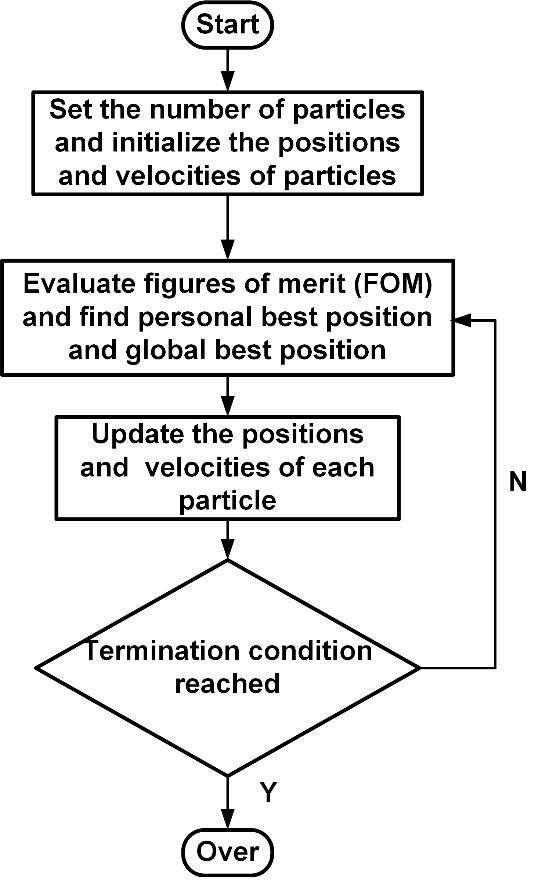


**Figure S1:** Flow chart of PSO algorithm.

**2. Calculation of Multipole Moment**

The multipole moments can be calculated by obtaining the polarization current distribution within the nanoparticle in Cartesian coordinates [1, 2]

$p\text{=}-\frac{1}{i\omega}\int jj_{0}\left(kr\right)\text{d}r+\frac{k^{2}}{10}\int\left\{\left[r\cdot j\right]r-\frac{1}{3}r^{2}j\right\}\frac{15J_{2}\left(kr\right)}{\left(kr\right)^{2}}\text{d}r$ (S2)

$m\text{=}\frac{1}{2}\int\left[r\times j\right]\frac{3J_{1}\left(kr\right)}{\left(kr\right)^{2}}\text{d}r$ (S3)

$\begin{matrix} \hat{Q}^{e}\text{=}-\frac{1}{i\omega}\int\left\{3\left(r\otimes j+j\otimes r\right)-2\left[r\cdot j\right]\hat{U}\right\}\frac{3J_{1}(kr)}{kr}dr \\ +6k^{2}\int\left\{5r\otimes r\left[r\cdot j\right]-\left(r\otimes j+j\otimes r\right)r^{2}-r^{2}\left[r\cdot j\right]\hat{U}\right\}\frac{J_{3}(kr)}{(kr)^{3}}dr \end{matrix}$ (S4)

$\hat{Q}^{m}\text{=}15\int\left\{\left[r\times j\right]\otimes r+r\otimes\left[r\times j\right]\right\}\frac{J_{2}(kr)}{\left(kr\right)^{2}}dr$ (S5)

where, **p**, **m**, ${\hat{\text{Q}}}^{\text{e}}$, ${\hat{\text{Q}}}^{\text{m}}$ are electric dipole moment, magnetic dipole moment, electric quadrupole moment and magnetic quadrupole moment in the Cartesian representation respectively, *J*_n_ denotes n^th^-order spherical Bessel function, $\hat{\text{U}}$ denotes the 3D unit tensor, and *k* is the wave number in the surroundings (air). The polarization current can be calculated by this equation:

$j=\text{-}i\omega\varepsilon_{0}(\varepsilon-\varepsilon_{d})E(r)$ (S6)

where *ω* is the angular frequency of the induced light, **E(r)** is the total electric ﬁeld inside the nanoparticles (a-Si), and *ε*_0_, *ε*, *ε*_d_ are the vacuum permittivity, relative permittivity of the nanoparticles and the surroundings (air) respectively. The scattering cross section of these multipole moments, i.e., the scattering power, can be calculated from the following equations [1]:

$C_{\text{sca}}^{p}=\frac{k^{4}}{6\pi\varepsilon_{0}^{2}\left|E_{\text{inc}}\right|^{2}}\sum_{\alpha} \left|p_{\alpha}\right|^{2}$ (S7)

$C_{\text{sca}}^{m}=\frac{k^{4}}{6\pi\varepsilon_{0}^{2}\left|E_{\text{inc}}\right|^{2}}\sum_{\alpha} \frac{\left|m_{\alpha}\right|}{c}^{2}$ (S8)

$C_{\text{sca}}^{Q^{e}}=\frac{k^{4}}{720\pi\varepsilon_{0}^{2}\left|E_{\text{inc}}\right|^{2}}\sum_{\alpha\beta} \left|kQ_{\alpha\beta}^{e}\right|^{2}$ (S9)

$C_{\text{sca}}^{Q^{m}}=\frac{k^{4}}{720\pi\varepsilon_{0}^{2}\left|E_{\text{inc}}\right|^{2}}\sum_{\alpha\beta} \left|\frac{kQ_{\alpha\beta}^{m}}{c}\right|^{2}$ (S10)

where *p_α_*, *m_α_*, $\text{Q}_{\text{αβ}}^{\text{e}}$, $\text{Q}_{\text{αβ}}^{\text{m}}$ are the electric and magnetic dipole moment, as well as the electric and magnetic quadrupole moment respectively, |**E**_inc_| is the electric field amplitude of the incident plane wave, *k* is the wavenumber in the surroundings, and *c* is the speed of light in vacuum.

The specific calculation process is as follows. First, we use finite difference time domain (FDTD) method to obtain the electric field distribution **E(r)** and the refractive index distribution *ε*(**r**) within a unit cell. In full wave simulations, periodic boundary conditions were implemented in the X and Y directions, while the Z direction was subject to perfectly matched layers (PMLs) to simulate the incidence of a plane wave from the substrate. After obtaining **E(r)** and *ε*(**r**) the polarization current distribution **j** can be calculated from Equation S6. With **j** the components of the multipole moment can be calculated numerically from Equation S2–S5. Once the components of the multipole moment are obtained, the scattering cross section of each multipole, i.e. the scattering power, can be calculated from Equation S7–S10. The calculated scattering power for HER structure is shown in Figure S2. To confirm the negligible contributions from higher order multipoles, the intensity of the octupole is also given in Figure S2. It is seen that the scattering intensity of the octupole is nearly two orders of magnitude lower than that of the first four multipoles within the operational band. This suggests that the octupole and even higher-order modes can be effectively neglected during the analysis based on multipole decomposition.
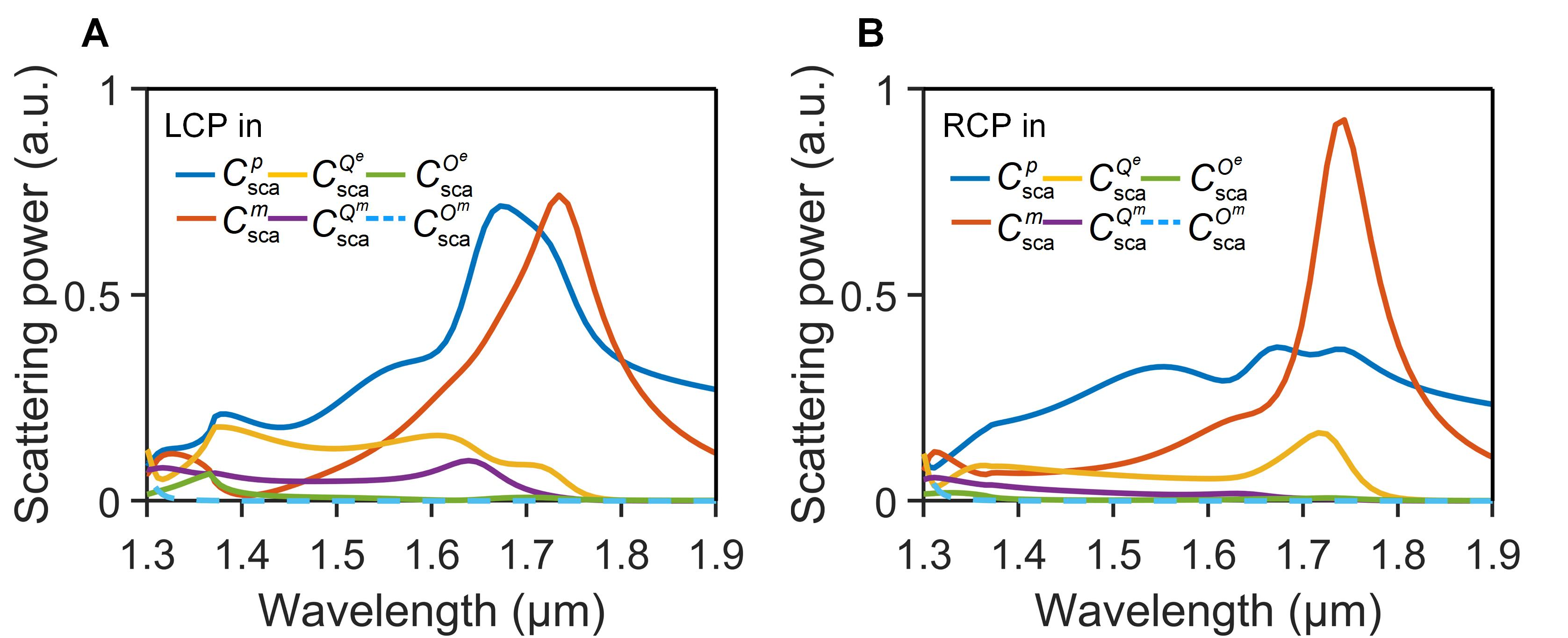


**Figure S2:** Scattering power of individual multipole components in HER structure up to octupole excited by (A) LCP and (B) RCP incidence.

**3. Vectorial field interference of multipoles in the reflection of HER structure under RCP incidence**

Figure S3A–D gives the plots of amplitude and phase of individual components, *F_i_* (∙) and their interaction processes under RCP incidence. The interaction among the components *F_i_* (∙) (*i* = 1–4) in $\text{E}_{\text{x}}^{\text{sc}}$ or *F_i_* (∙) (*i* = 5–8) in $\text{E}_{\text{y}}^{\text{sc}}$ can be understood by the two-stage interference of these components In $\text{E}_{\text{x}}^{\text{sc}}$, the phase difference between *F*_1_ and *F*_3_ is ~90°, resulting in an interferometric field *F*_1_+*F*_3_. Similarly, the phase difference between *F*_2_ and *F*_4_ is ~90° resulting in an interferometric field *F*_2_+*F*_4_. As a result, the fields of *F*_1_+*F*_3_ and *F*_2_+*F*_4_ are almost in phase, i.e., the phase difference between *F*_1_+*F*_3_ and *F*_2_+*F*_4_ is less than 90°, which results in constructive interference. The amplitude and phase of the first-stage interference, *F*_1_+*F*_3_ and *F*_2_+*F*_4_, are plotted with a solid green and a blue line, respectively, in Figure S3A and B. The amplitude after the second-stage interference between the fields *F*_1_+*F*_3_ and *F*_2_+*F*_4_ is plotted with a solid black line in Figure S3A. It is seen that the overall amplitude of $\text{E}_{\text{x}}^{\text{sc}}$ after the 2-stage interference becomes high and flat within the whole wavelength band of interest (grey shaded area). Similarly, a two-stage constructive interference among the four components, *F_i_* (∙) (*i* = 5–8) of $\text{E}_{\text{y}}^{\text{sc}}$ results in an overall field *F*_5_+*F*_6_+*F*_7_+*F*_8_ with a high amplitude, as depicted in Figure S3C and D. The intensity of the far field of reflection can thus be calculated by ($\text{ }{{\text{|}\text{E}}_{\text{x}}^{\text{sc}}\text{|}}^{\text{2}}\text{ + }{{\text{|}\text{E}}_{\text{y}}^{\text{sc}}\text{|}}^{\text{2}}$), which will result in a high reflection in the case of RCP incidence.


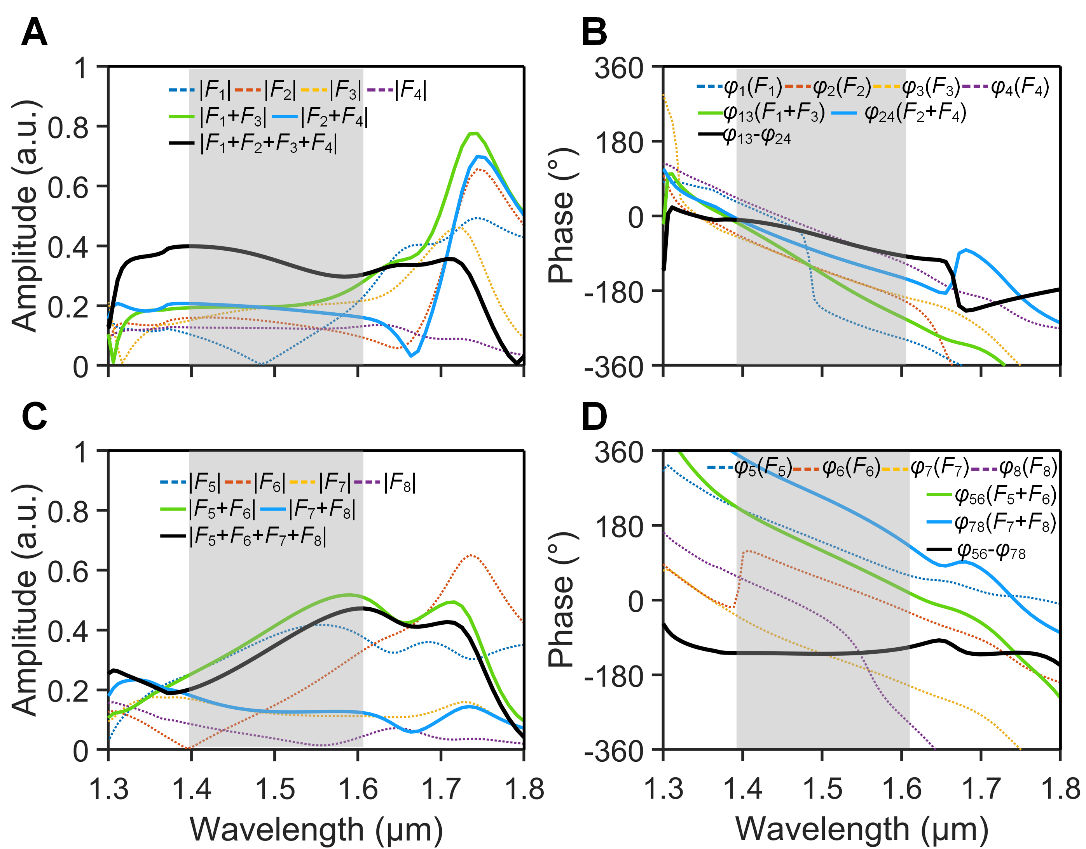


**Figure S3:** Vectorial field interference of multipoles in the reflection of HER structure under RCP incidence. (A, C) Amplitudes of the four components of $\text{E}_{\text{x}}^{\text{sc}}$ and $\text{E}_{\text{y}}^{\text{sc}}$, respectively, as well as the amplitudes formed by the first-stage and second-stage interference. (B, D) The corresponding phases of different components of $\text{E}_{\text{x}}^{\text{sc}}$ and $\text{E}_{\text{y}}^{\text{sc}}$, and those after the first-stage and second-stage interference.

Figure S4 shows the intensity of the far field of reflection (${\text{|}\text{E}^{\text{sc}}\text{|}}^{\text{2}}\text{ =}{\text{ |}\text{E}_{\text{x}}^{\text{sc}}\text{|}}^{\text{2}}\text{ + }{\text{|}\text{E}_{\text{y}}^{\text{sc}}\text{|}}^{\text{2}}$) when LCP and RCP incident the HER structure, respectively. It can be seen within the working band of HER, denoted by the grey region in the figure, the reflected field intensities of the RCP incidence are noticeably higher compared to those of the LCP incidence, which is consistent with the result in Figure 2D. This finding provides a convincing explanation for the broadband CD exhibited by the proposed chiral metasurface.


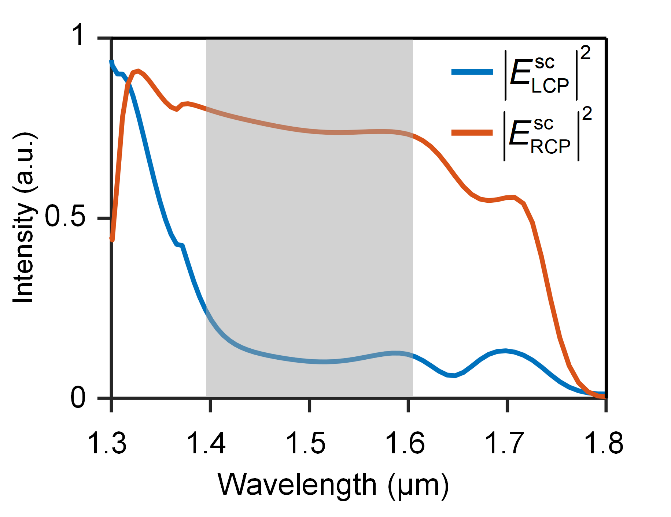


**Figure S4:** Scattering CD spectrum for the structure HER based on semi-quantitative multipoles model.

**4. Vectorial field interference of multipoles in the UBB structure**

Due to the structural similarity, the interaction among the multipoles of the UBB structure is the same as that of the HER structure. The detailed vectorial field interference of multipoles in the reflection of UBB structure under LCP and RCP incidence are given in Figure S5 and Figure S6, respectively.


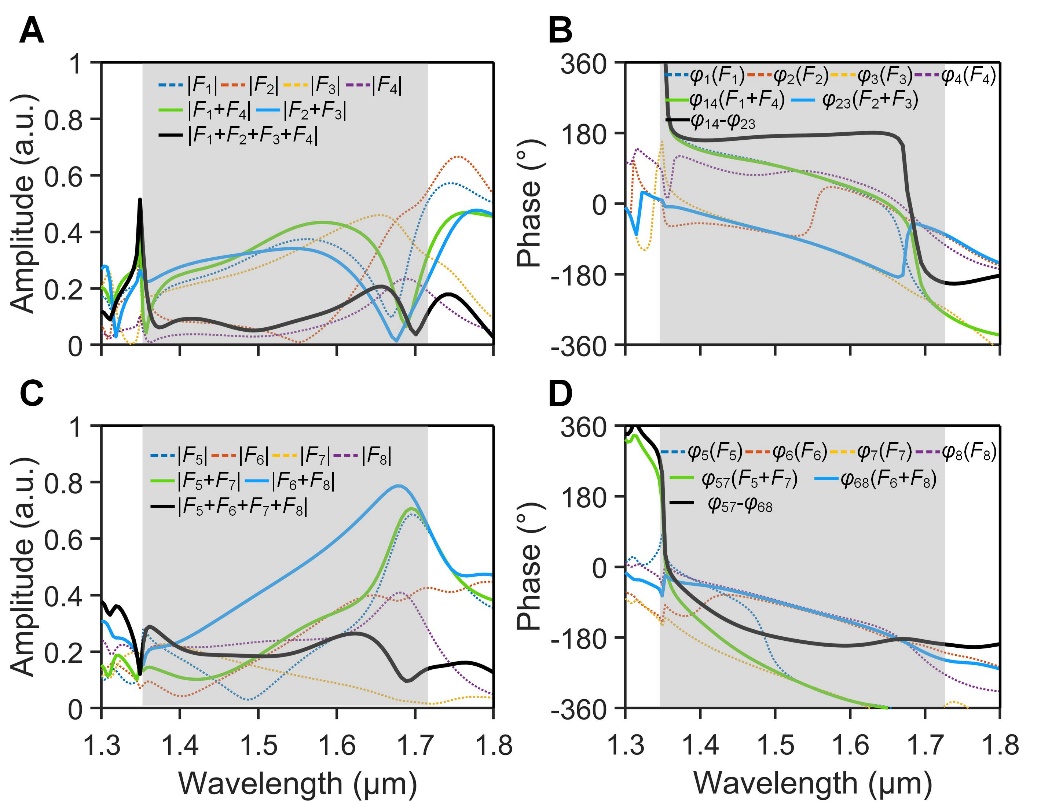


**Figure S5:** Vectorial field interference of multipoles in the reflection of UBB structure under LCP incidence. (A, C) Amplitudes of the four components of $\text{E}_{\text{x}}^{\text{sc}}$ and $\text{E}_{\text{y}}^{\text{sc}}$, respectively, as well as the amplitudes formed by the first-stage and second-stage interference. (B, D) The corresponding phases of different components of $\text{E}_{\text{x}}^{\text{sc}}$ and $\text{E}_{\text{y}}^{\text{sc}}$, and those after the first-stage and second-stage interference.


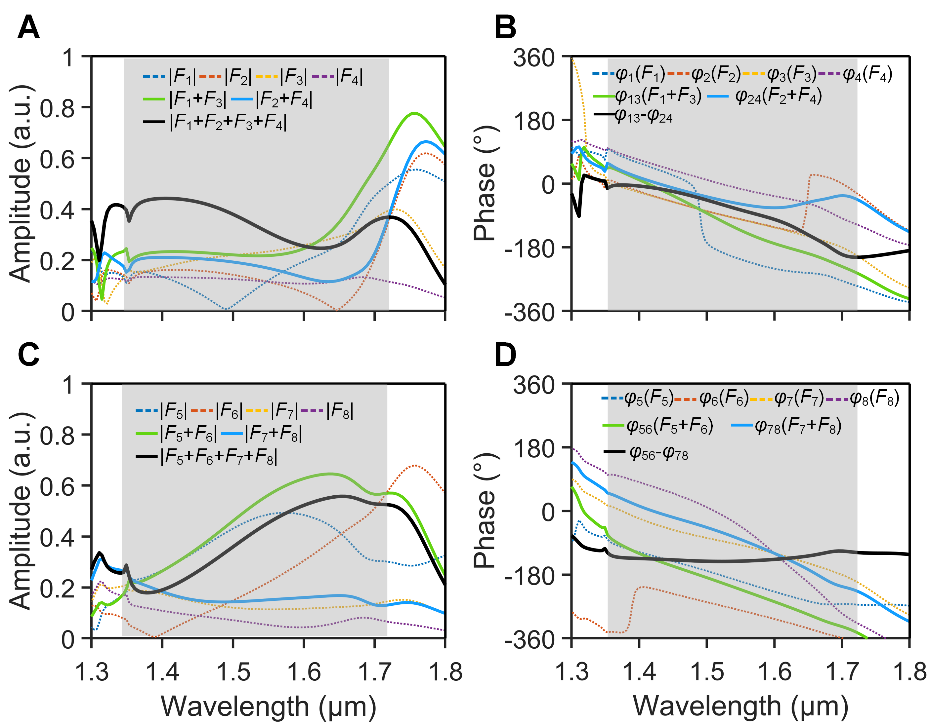


**Figure S6:** Vectorial field interference of multipoles in the reflection of UBB structure under RCP incidence. (A, C) Amplitudes of the four components of $\text{E}_{\text{x}}^{\text{sc}}$ and $\text{E}_{\text{y}}^{\text{sc}}$, respectively, as well as the amplitudes formed by the first-stage and second-stage interference. (B, D) The corresponding phases of different components of $\text{E}_{\text{x}}^{\text{sc}}$ and $\text{E}_{\text{y}}^{\text{sc}}$, and those after the first-stage and second-stage interference.

Figure S7 shows the intensity of the far field of reflection (${\text{|}\text{E}^{\text{sc}}\text{|}}^{\text{2}}\text{ =}{\text{ |}\text{E}_{\text{x}}^{\text{sc}}\text{|}}^{\text{2}}\text{ + }{\text{|}\text{E}_{\text{y}}^{\text{sc}}\text{|}}^{\text{2}}$) when LCP and RCP incident the UBB structure, respectively. It can also be seen within the working band of UBB, denoted by the grey region in the figure, the reflected field intensities of the RCP incidence are noticeably higher compared to those of the LCP incidence, which is consistent with the result in Figure 2H.


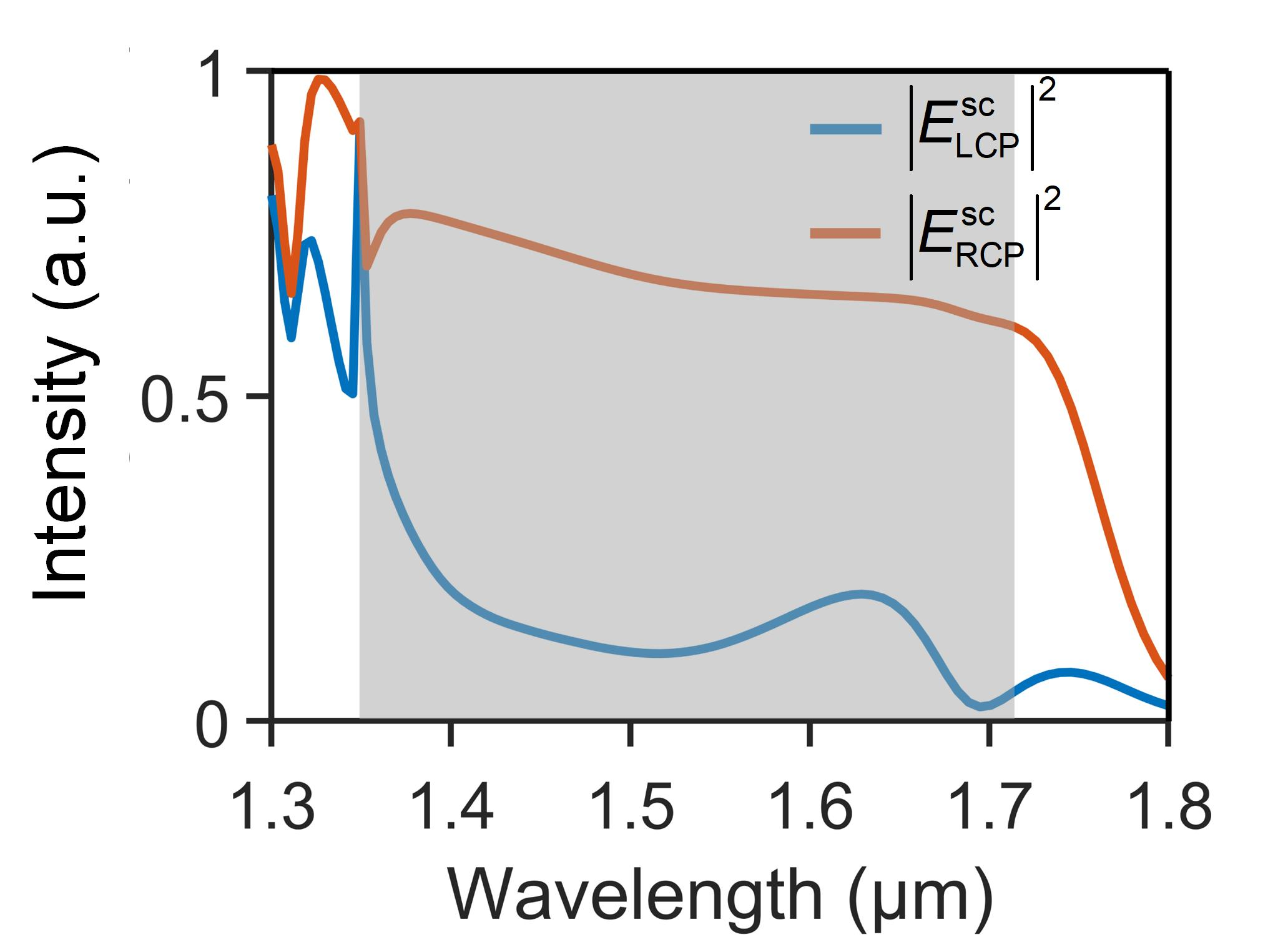


**Figure S7:** Scattering CD spectrum for the structure UBB based on semi-quantitative multipoles model.

**5. Experimental measurement setup**

The optical setup for the measurement of chiral characteristics is depicted in Figure S8. A supercontinuum laser (Fianium LLTF Contrast) emits wavelength-tunable monochromatic light, which is then converted to circularly polarized light by an attenuator (A, NDC-50S-1M), an achromatic linear polarizer (P, Thorlabs, WP25H) and an achromatic quarter-wave, (QWP, AQWP10M-1600). The circularly polarized light is then focused onto the sample surface by a near-infrared double-glued achromatic lens (L, Thorlabs, AC-254-075-C, f = 75 mm), with a focal area smaller than that of the metasurface. The transmitted light from the chiral structure array passes through a microscopic objective (O, Mitutoyo, MY50X-825, 50×) and is imaged onto a NIR CCD camera (XENICS, XEVA-1.7-320, 320×256). The energy of the transmitted light is then determined by taking a photograph and normalized to the transmission energy of the incident light passing through a bare glass substrate.


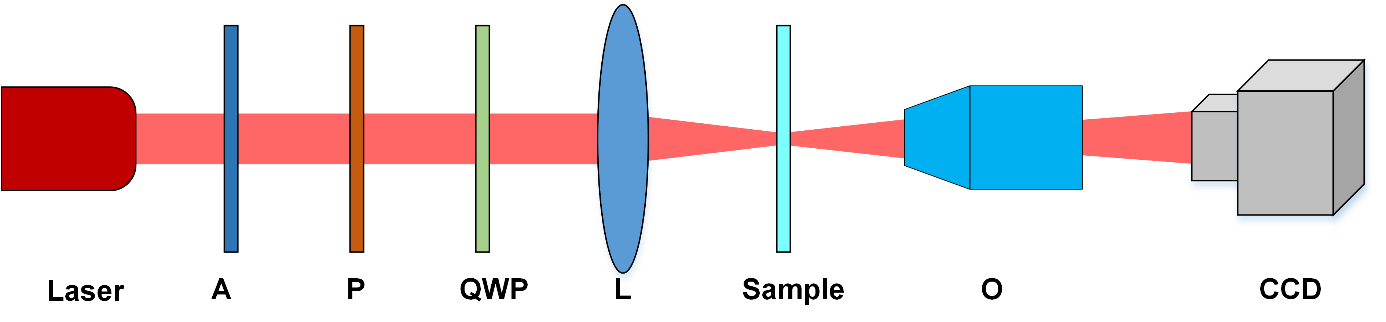


**Figure S8:** Schematic of chiral spectrum measurement.

**6. Comparison of CD/PER and Bandwidth of Chiral Structures**

**Table S1.** Comparison of CD/PER and Bandwidth of Chiral Structures

| Refs | Working band | Materials and 2D/3D | Average CD  Theo./Exp. | Maximum PER  Theo./Exp. |
| --- | --- | --- | --- | --- |
| [3] | 3.5–7.5 μm | Gold, 3D | ∼0.7 (Exp.) | ∼9 dB (Exp.) |
| [4] | 30–90 THz | Gold, 3D | ∼0.5 (Exp.) | <10 dB (Exp.) |
| [5] | 500–800 nm | Gold, 3D | ∼0.7 (Theo.) | ∼10 dB (Theo.) |
| [6] | 1.55 μm | Silicon, 3D | ∼0.7 (Exp.) | <10 dB (Exp.) |
| [7] | 1.20–1.50 μm | Silver, 2D | ∼0.7 (Exp.) | <10 dB (Exp.) |
| [8] | 1.49–1.61 μm | a-Si, 2D | ∼0.5 (Exp.) | <10 dB (Exp.) |
| [9] | 540 nm | TiO_2_, 2D | 0.8 (Exp.) | ∼10 dB (Exp.) |
| [10] | 870 nm | Si_3_N_4_, 2D | ∼0.4 (Exp.) | < 10 dB (Exp.) |
| [11] | 550–700 nm | a-Si, 2D | ∼0.5 (Exp.) | <10 dB (Exp.) |
| [12] | 1 THz | Silicon, 2D | ∼0.4 (Exp.) | <10 dB (Exp.) |
| [13] | 747 nm | Perovskite, 2D | 0.16 (Exp.) | <10 dB (Exp.) |
| This work | 1.35–1.72 μm | a-Si, 2D | >0.9 (Exp.)@1.39–1.61 μm  >0.8(Exp.)@1.35–1.65 μm | 19.7 dB (Exp.)  17.5 dB (Exp.) |

Table S1 lists the recent advances and comparison of the performances of chiral structures in terms of working wavelength band, bandwidth, CD, and PER. It is seen that most of structures worked at a single wavelength or in a narrow wavelength range. For the reported broadband devices, the CD and PER are compromised over the operating band. Our proposed structure can simultaneously have a high CD and PER over a broadband wavelength range, which has great potential applications in various polarimetric imaging and detections.

**References**

[1] R. Alaee, C. Rockstuhl, and I. Fernandez-Corbaton, “An electromagnetic multipole expansion beyond the long-wavelength approximation,” *Opt. Commun.*, vol. 407, pp 17-21, 2018, https://doi.org/10.1016/j.optcom.2017.08.064.

[2] T. Hinamoto, and M. Fujii, “Menp: An open-source matlab implementation of multipole expansion for nanophotonics,” *OSA Continuum*, vol. 4, no. 5, pp 1640-1648, 2021, https://doi.org/10.1364/OSAC.425189.

[3] J. K. Gansel, M. Thiel, M. S. Rill, et al., “Gold helix photonic metamaterial as broadband circular polarizer,” *Science*, vol. 325, no. 5947, pp. 1513-1515, 2009, https://doi.org/10.1126/science.1177031.

[4] J. K. Gansel, M. Latzel, A. Frolich, J. Kaschke, M. Thiel, and M. Wegener, “Tapered gold-helix metamaterials as improved circular polarizers,” *Appl. Phys. Lett.*, vol. 100, no. 10, p. 101109, 2012, https://doi.org/10.1063/1.3693181.

[5] Y. Zhao, M. A. Belkin, and A. Alu, “Twisted optical metamaterials for planarized ultrathin broadband circular polarizers,” *Nat. Commun.*, vol. 3, p. 870, 2012, https://doi.org/10.1038/ncomms1877.

[6] K. Tanaka, D. Arslan, S. Fasold, et al., “Chiral bilayer all-dielectric metasurfaces,” *ACS Nano*, vol. 14, no. 11, pp. 15926-15935, 2020, https://doi.org/10.1021/acsnano.0c07295.

[7] W. Li, Z. J. Coppens, L. V. Besteiro, W. Wang, A. O. Govorov, and J. Valentine, “Circularly polarized light detection with hot electrons in chiral plasmonic metamaterials,” *Nat. Commun.*, vol. 6, p. 8379, 2015, https://doi.org/10.1038/ncomms9379.

[8] J. Hu, X. Zhao, Y. Lin, et al., “All-dielectric metasurface circular dichroism waveplate,” *Sci. Rep.*, vol. 7, p. 41893, 2017, https://doi.org/10.1038/srep41893.

[9] A. Y. Zhu, W. T. Chen, A. Zaidi, et al., “Giant intrinsic chiro-optical activity in planar dielectric nanostructures,” *Light: Sci. Appl.*, vol. 7, p. 17158, 2018, https://doi.org/10.1038/lsa.2017.158.

[10] B. Semnani, J. Flannery, R. Al Maruf, and M. Bajcsy, “Spin-preserving chiral photonic crystal mirror,” *Light: Sci. Appl.*, vol. 9, no. 1, p. 23, 2020, https://doi.org/10.1038/s41377-020-0256-5.

[11] H. S. Khaliq, I. Kim, A. Zahid, et al., “Giant chiro-optical responses in multipolar-resonances-based single-layer dielectric metasurfaces,” *Photonics Res.*, vol. 9, no. 9, pp. 1667-1674, 2021, https://doi.org/10.1364/PRJ.424477.

[12] J. Li, J. Li, C. Zheng, et al., “Lossless dielectric metasurface with giant intrinsic chirality for terahertz wave,” *Opt. Express*, vol. 29, no. 18, pp. 28329-28337, 2021, https://doi.org/10.1364/OE.430033.

[13] G. Long, G. Adamo, J. Tian, et al., “Perovskite metasurfaces with large superstructural chirality,” *Nat. Commun.*, vol. 13, no. 1, p. 1551, 2022, https://doi.org/10.1038/s41467-022-29253-0.
